# Effects of different proportions of soft rock additions on organic carbon pool and bacterial community structure of sandy soil

**DOI:** 10.1038/s41598-021-84177-x

**Published:** 2021-02-25

**Authors:** Wan-ying Li, Zhen Guo, Juan Li, Ji-chang Han

**Affiliations:** 1 College of Land Engineering, Chang’an University, Xi’an, 710064 Shaanxi China; 2 Shaanxi Provincial Land Engineering Construction Group Co., Ltd., Xi’an, 710075 Shaanxi China; 3 Institute of Land Engineering and Technology, Shaanxi Provincial Land Engineering Construction Group Co., Ltd., Xi’an, 710021 Shaanxi China

**Keywords:** Environmental sciences, Solid Earth sciences, Engineering, Chemical physics

## Abstract

The sandy soil leaks water and fertilizer, and the ecological degradation is serious. The structural characteristics of soft rock and sandy soil are complementary, and the improvement of sandy soil by adding soft rock is of great significance to improve soil fertility, restore biodiversity, and maintain sustainable development of the Mu Us sandy land region. In this study, total organic carbon (TOC), particulate organic carbon (POC), dissolved organic carbon (DOC), easily oxidized organic carbon (ROC), microbial biomass carbon (SMBC), bacterial community structure and crop yield were examined using soft rock:sand volume ratios of 0:1 (CK), 1:5 (C1), 1:2 (C2) and 1:1 (C3). Our results indicated that, compared with the CK treatment, TOC (9.66–22.34%), POC (85.65–120.41%) and ROC (114.12–192.31%) noticeably increased in C1, C2 and C3 treatments; SMBC in treatment C3 increased by 42.77%. The three dominant bacteria in the soil (*Proteobacteria*, *Actinobacteria* and *Chloroflexi*), as well as *Proteobacteria* abundance, greatly declined in the treatments with the addition of soft rock. *Pseudarthrobacter* was the dominant Genus in all treatments, having an abundance between 11.83 and 19.33%. Bacterial diversity, richness and evenness indices all recorded an increase under the treatments. POC, TOC and SMBC recorded the most significant effects on the bacterial community structure. The largest increases in wheat and corn yields were recorded in the C2 treatment (16.05% and 16.30%), followed by the C1 treatment (8.28% and 8.20%, respectively). Our findings indicate that a soft rock:sand ratio between 1:5 and 1:2 recorded the most improvement in the sandy soil environment.

## Introduction

Due to the impact of human activities, such as transitional grazing and transitional mining, various degrees and types of degradation have been recorded in areas with sandy vegetation and soil^1^. Degradation of sandy land seriously threatens the sustainable development of animal husbandry and crop industry in arid and semi-arid areas, affecting socio-economic status and ecological environment^2^. In China, desertification is one extreme manifestation of land degradation; desertification covers an area ~ 3.59 × 10^5^ km^2^, accounting for 3.74% of the land area^3^. During the process of desertification, soil becomes coarse, its structure becomes more loose and its nutrient content declines, affecting its water retention, compactness and adhesion to mineral elements^4^. A reduction of soil nutrients and production potential caused by soil desertification is directly affecting vegetation degradation. As a result, the structure and function of sandy land are unbalanced, seriously restricting ecosystem restoration^5^. Therefore, except for improving the sandy soil quality, the realization of resource utilization and biological restoration of desertified land, while achieving economic benefit, environmental protection and energy saving, is of great significance to reduce regional soil erosion and restore regional ecology..

The soil organic carbon (SOC) pool, being mainly formed by the decomposition of organic matter such as animal and plant residues and root exudates, is an essential index for evaluating the degree of land degradation^6^. Although SOC is not important for plant nutrient cycling, it plays an important role in maintaining and consolidating soil structure^7^. By analyzing the different component types of carbon, Yang et al*.*^8^ concluded that fast-changing activated carbon and light group carbon could be used as indicators of change for the soil carbon pool. They also recorded that closed and accumulative micro-aggregate organic carbon, inert mineral particle combined carbon and recombined carbon had a high level of stability, thus being stored in the soil for a long time. Bajgai et al*.*^9^ highlighted that SOC is a complex composed of components with different activities, turnover rates and functions, and soil microorganisms play a vital role in SOC turnover and the carbon pool balance. Soil microorganisms are not only the driving force of soil organic matter circulation and transformation, they are also the main body that promotes and maintains material circulation and energy flow^10,11^. Groups of soil microorganisms that are essential in an ecosystem can be identified using soil microorganism community diversity^12,13^. For example, Janzen et al*.*^14^ highlighted that easily oxidized organic carbon (ROC) content had a high correlation with soil respiration rate, indicating that ROC was a carbon and energy source of soil microbial physiological activities, having a significant impact on microbial community structure. Tang et al*.*^15^ also showed that soil microorganisms decompose soil coarse-grained organic carbon into fine-grained organic carbon; with an increase of microbial diversity, the cohesive effect of aggregates also increases, thus promoting an increase in organic carbon content in the soil. Findings by Dong et al.^16^ highlighted that the content of active components in a soil, such as microbial biomass carbon (SMBC) and dissolved organic carbon (DOC), were substantially improved by the addition of exogenous material, thereby increasing SOC content as well as providing an available substrate for soil microorganisms.

The Mu Su desert is one of the four largest sandy lands in China, covering an area of 42,200 square kilometers. The area is widely distributed with two kinds of natural resources, soft rock and sand, characterized by serious soil erosion and loose texture, low nutrient content and poor soil structure^17^. The soft rock has a loose structure and is easy to weather, calcium montmorillonite and other mineral components in which will disintegrate when exposed to water, and the content of clay and silt particles is as high as 90%. On the other hand, the sandy soil is basically non-structural and has poor resistance to wind erosion, and the particle content is mainly composed of sand, which is more than 95%^18,19^. Based on soft rock and sand properties, Han et al.^18^ highlighted that a soft rock:sand ratio between 1:5 and 1:1 can promote crop growth, findings that have been widely used in current management decisions. Sun et al.^19^ pointed out that soft rock has great potential in improving crop yield in Mu Us sandy land, and the increase in crop yield depends on the improvement of sandy soil fertility and biodiversity. Although hydraulic properties, physical structure and adsorption of composite soil in early stages were examined^17–20^, the relationship between different organic carbon components and microorganisms in mixed soils have not be investigated. The aim of this study, therefore, was to: (1) clarify the effects of different proportions of soft rock additions on SOC components and crop yield; (2) identify the effects of different proportions of soft rock additions on bacterial community structure and diversity; and (3) identify the dominant carbon pool components that affect the bacteria community structure.

## Results and discussion

### Soil total organic carbon (TOC)

Results for total organic carbon (TOC) content (Table 1) recorded as increase with different proportions of soft rock mixed with sand. TOC in C1, C2 and C3 were 18.73%, 9.66% and 22.34% greater than the control, respectively; TOC in CK was 2.99 g kg^-1^. Because the sandy soil structure was loose, the content of sand grains was 95% or higher, and the carbon sequestration capacity was poor^21^. Due to the large number of fine particles in the soft rock, addition of this material can balance the lack of clay particles in the sandy soil^18^. Over time, the higher content of silt and clay in the soft rock played an important role, resulting in an enhancement of the soil’s ability to retain water and fertilizers, thus being beneficial to plant growth and the accumulation of plant residues, thereby promoting the accumulation of organic carbon^17^. These changes were identified in our experiment with treatment C3 recording the highest organic carbon content.

**Table 1:** Organic carbon components in the mixed soils with different proportions of soft rock and sand.

Treatments	TOC (g kg^-1^)	POC (g kg^-1^)	DOC (mg kg^-1^)	ROC (mg kg^-1^)	SMBC (mg kg^-1^)
CK	2.99 ± 0.68 a	1.32 ± 0.12 b	0.75 ± 0.07 b	0.26 ± 0.05 b	28.15 ± 0.11 b
C1	3.55 ± 0.45 a	2.79 ± 0.42 a	0.84 ± 0.01 a	0.66 ± 0.20 a	38.17 ± 0.01 ab
C2	3.28 ± 0.16 a	2.46 ± 0.18 a	0.77 ± 0.01 b	0.76 ± 0.06 a	36.86 ± 0.41 ab
C3	3.66 ± 0.33 a	2.92 ± 0.09 a	0.84 ± 0.03 a	0.56 ± 0.09 a	40.20 ± 0.99 a

Mean ± SD, different lowercase letters in the same column indicate 5% difference between treatments.

CK, the volume ratio of soft rock to sand is 0:1; C1, the volume ratio of soft rock to sand is 1:5; C2, the volume ratio of soft rock to sand is 1:2; C3, the volume ratio of soft rock to sand is 1:1; *TOC* soil total organic carbon; *POC* particulate organic carbon; *DOC* dissolved organic carbon; *ROC* oxidized organic carbon; *SMBC* soil microbial biomass carbon.

### Different organic carbon components

SOC consists of components with different levels of stability, including active organic carbon and inert organic carbon. Among these, active organic carbon is derived from the decomposition of plant litter, root exudates, hydrolysis of soil organic matter, soil microorganisms and their metabolites, playing a vital role in soil fertility and changes in soil carbon storage^22^. POC is a semi-decomposed organic matter component derived from animal and plant residues, considered to be a sensitivity index of soil management affecting SOC dynamics in addition to SOC active components^9^. Our results indicated that C1, C2 and C3 treatments vastly increased POC content (Table 1) due to organic carbon content of the soft rock being higher than that of the sandy soil; the addition of soft rock improved the structure of the sandy soil. The proportion of POC to TOC varied from 46.22% to 80.94% (Table 2), indicating that the addition of soft rock increased the fine particle content of the sandy soil and increased the content of sandy soil micro-aggregates, resulting in POC content to account for the largest proportion^18,23^. DOC is closely related to soil microaggregates, being one of the active forms of organic carbon that is sensitive to the response of soil management measures^24^. DOC content in our study were highest in treatments C1 and C3, being about 12.00% higher than those of the control; DOC content in C2 was not notably different from the CK treatment (Table 1). Although the proportion of DOC in TOC ranged from 23.38 to 26.57%, and gradually decreased with an increase in the proportion of soft rock, no significant differences were recorded (P > 0.05) (Table 2). This result was associated to the gradual increase in mineral elements in the compound soil and a gradual decrease of carbohydrates as the proportion of soft rock increased^17^. It is also possible that the soil structure in treatments C3 and C1 had better contact, reduced porosity, and increased soil moisture content, leading to a release of DOC into soil water^25^.

**Table 2:** Percentage of different organic carbon components in soil total organic carbon (%).

Treatments	POC/TOC	DOC/TOC	ROC/TOC	SMBC/TOC
CK	46.22 ± 11.11 b	26.57 ± 7.57 a	8.93 ± 1.51 c	1.00 ± 0.30 a
C1	78.84 ± 2.50 a	24.25 ± 4.04 a	18.20 ± 2.31 ab	1.10 ± 0.20 a
C2	76.14 ± 15.49 a	23.64 ± 2.67 a	23.53 ± 4.82 a	1.14 ± 0.19 a
C3	80.94 ± 11.35 a	23.38 ± 4.30 a	15.65 ± 4.31 b	1.10 ± 0.20 a

Mean ± SD, different lowercase letters in the same column indicate 5% difference between treatments.

CK, the volume ratio of soft rock to sand is 0:1; C1, the volume ratio of soft rock to sand is 1:5; C2, the volume ratio of soft rock to sand is 1:2; C3, the volume ratio of soft rock to sand is 1:1; *TOC* soil total organic carbon; *POC* particulate organic carbon; *DOC* dissolved organic carbon; *ROC* oxidized organic carbon; *SMBC* soil microbial biomass carbon.

ROC composition was mainly a linear hydrocarbon compound that can be oxidized by potassium permanganate within a certain period of time, and its content level is largely affected by human factors such as farming. Zhang and Han^26^ showed that long-term application of chemical fertilizers could significantly increase DOC content in black soil. Results from our study indicated that under the condition of long-term application of chemical fertilizers, the addition of different proportions of soft rock could greatly increase DOC content in the sandy soil (115.38–192.31%) compared with the CK treatment (Table 1). This result may be due to rich hydrocarbons present in the soft rock^18,26^. In addition, our results indicated that the ratio of ROC to TOC was higher in C2 and C1 treatments, being noticeably higher than that in C3 and CK (Table 2). This result is associated to soft rock being an essential clay mineral, and its cemented substances can promote the formation of sandy soil organic carbon protection mechanisms, such as the physical protection of aggregates, the chemical protection of iron and aluminum, and the effects of microbial action. These mechanisms will gradually result in the transformation of ROC activated carbon to inert carbon, thus accounting for the smaller ROC/TOC result in C3^17,23,25^. SMBC is predominantly organic carbon combined with soil microorganisms, such as fungi and bacteria, and it is closely related to the conversion between soil organic carbon^10^. Trend results for SMBC and TOC changes in our study were basically the same among the treatments, specifically presented as C3 > C1 > C2 > CK (Table 1), indicating no significant differences were recorded in the ratio of SMBC to TOC among the treatments (Table 2). Because TOC is the substrate of soil microbial mineralization, and SMBC is one of the important factors affecting SOC mineralization^12^. Under the action of small biological cycles, soil microbial activity increases with the conversion of TOC substrate to active organic carbon also SMBC is an important carbon source that constitutes soil humus, thus SMBC and TOC have the same changing trend^27^.

### Bacterial community composition based on phylum level

**Figure 1:**
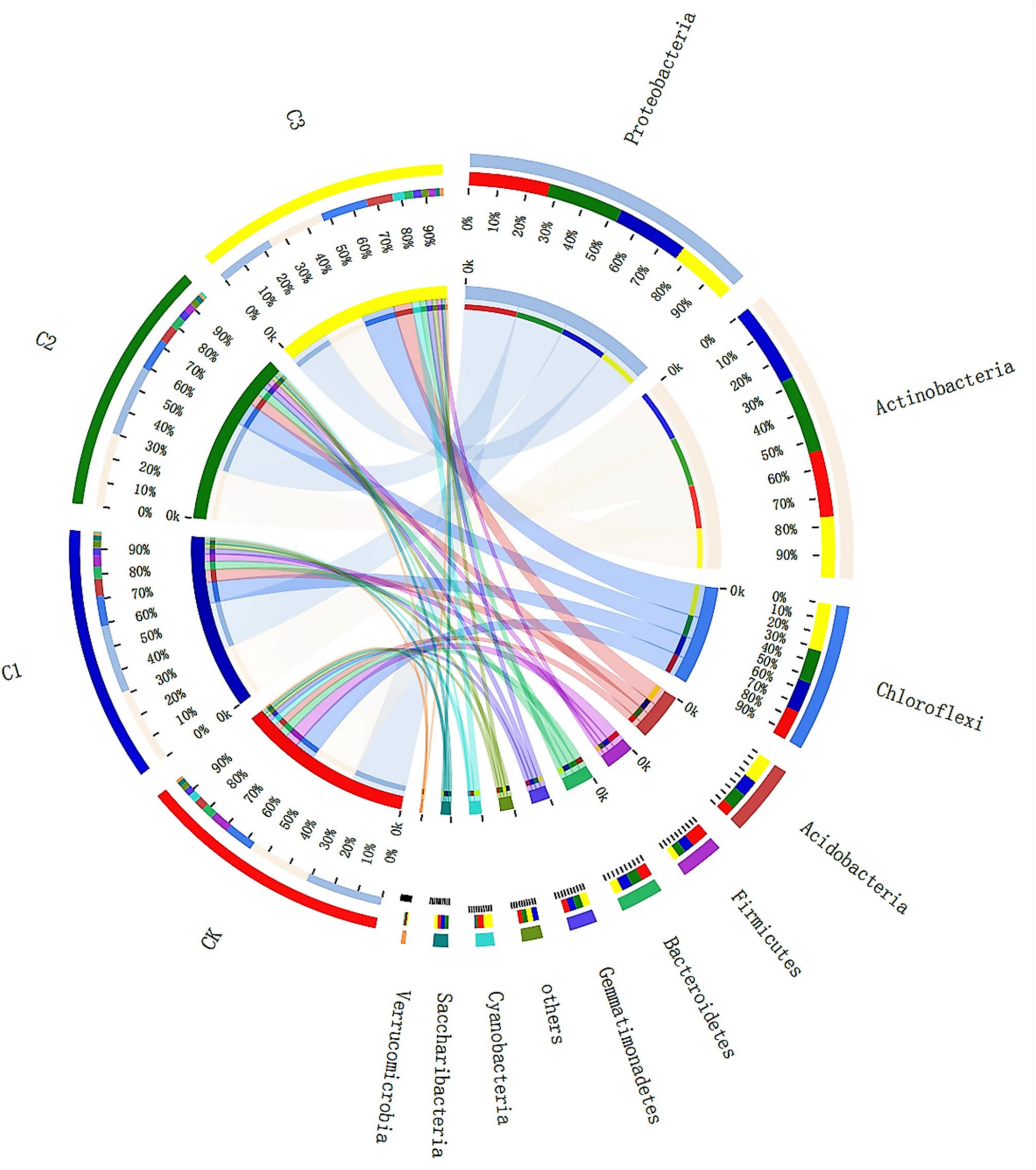
The abundance of soil bacteria on the Phylum level in the soft rock:sand compound soil. The image was created with the software Circos-0.67–7 (http://circos.ca/). CK, the volume ratio of soft rock to sand is 0:1; C1, the volume ratio of soft rock to sand is 1:5; C2, the volume ratio of soft rock to sand is 1:2; C3, the volume ratio of soft rock to sand is 1:1.

Our results indicate that bacterial abundance changed with the addition of soft rock. The dominant bacteria in all treatments were *Proteobacteria*, *Actinobacteria* and *Chloroflexi*, accounting for 67.87% to 74.12% of total bacterial abundance (Fig. 1). The abundance of *Proteobacteria* in soft rock treatments was noticeably lower than the abundance in the CK treatment; the abundance of *Actinobacteria* in C1 and C2 treatments was significantly higher than that in the CK treatment; and the abundance of *Chloroflexi* recorded a significant increase in C3. *Proteobacteria* and *Actinobacteria* have been recorded to belong to aerobic bacteria, and *Chloroflexi* belongs to facultative anaerobic bacteria. With the continuous increase in soft rock, crust phenomenon occurs in the sandy soil structure, resulting in a decrease in porosity and a decrease in soil oxygen content^25,28^. The abundance of *Acidobacteria* and *Gemmatimonadetes* gradually increased as the volume of soft rock increased, recording an increase of 56.23–71.06% (P < 0.05) and 8.47–20.68%, respectively. These increases may be associated to an increase in organic colloids in the compound soil of soft rock, especially polysaccharides which can provide abundant energy for microorganisms^13,17^. As the proportion of soft rock increased, the abundance of *Firmicutes* and *Bacteroidetes* decreased, recording declines of 44.20–65.81% (P < 0.05) and 0.19–24.60%, respectively. These declines in abundance may also be associated to *Firmicutes* and *Bacteroidetes* being aerobic bacteria. Compared with the CK treatment, C1 and C2 had a noticeable reduction in *Cyanobacteria* abundance; the abundance of *Saccharibacteria* and *Verrucomicrobia* did not differ considerably between treatments. Other species (< 1% abundance in each treatment) accounted for 2.22–2.94% of total bacterial abundance.

### Bacterial community composition based on genus level

**Figure 2:**
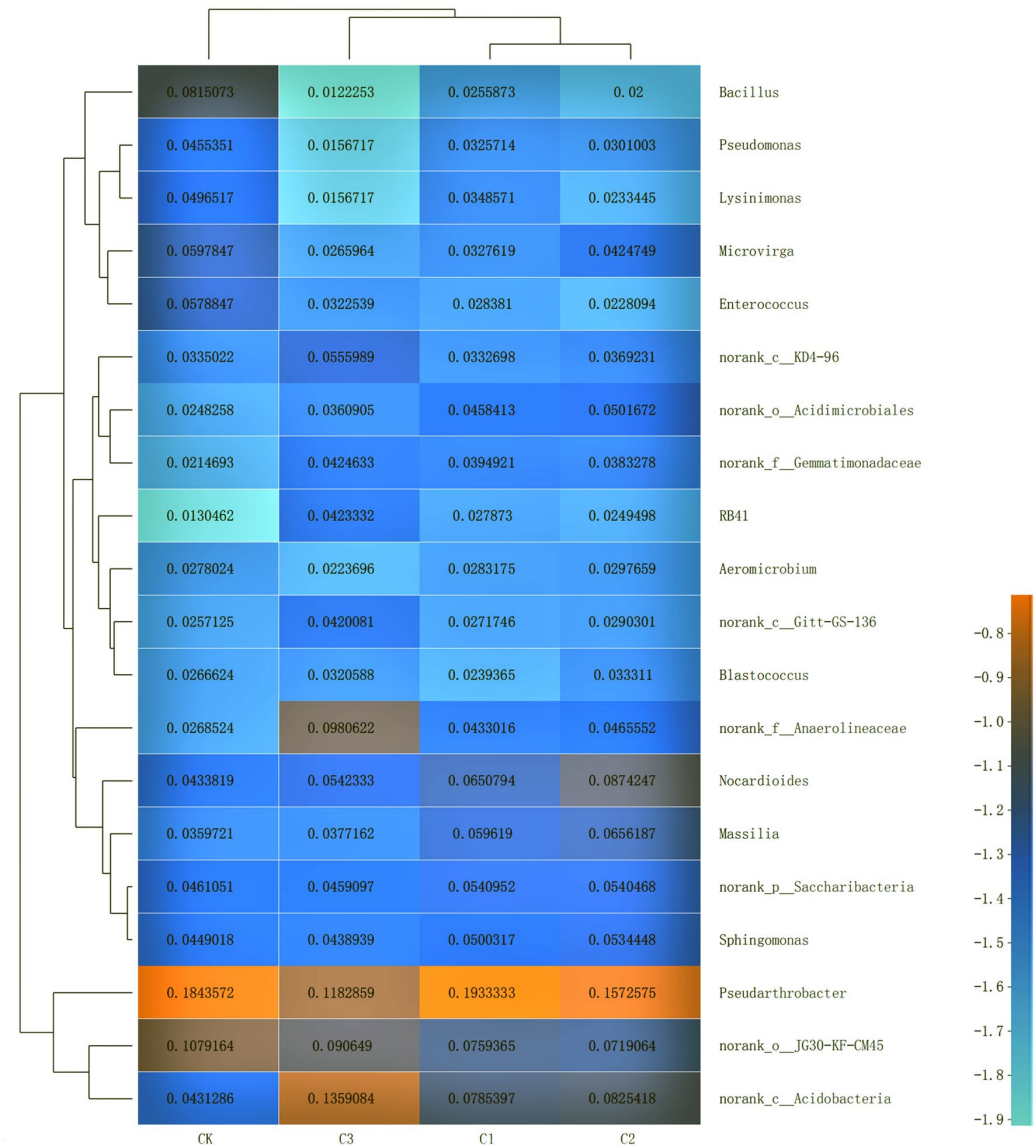
The abundance of soil bacteria on the Genus level in the soft rock:sand compound soil. CK, the volume ratio of soft rock to sand is 0:1; C1, the volume ratio of soft rock to sand is 1:5; C2, the volume ratio of soft rock to sand is 1:2; C3, the volume ratio of soft rock to sand is 1:1.

On the Genus level, the three dominant bacteria in CK treatment were *Pseudarthrobacter*, *norank_o__JG30-KF-CM45* and *Bacillus*. *Pseudarthrobacter*, *norank_c__Acidobacteria* and *norank_o__JG30-KF-CM45* were dominant in C1; *Pseudarthrobacter*, *Nocardioides* and *norank_c__Acidobacteria* were dominant in C2; and *norank_c__Acidobacteria*, *Pseudarthrobacter* and *norank_f__Anaerolineaceae* were dominant in C3 (Fig. 2). Our results indicate that *Bacillus*, *norank_f__Anaerolineaceae*, *Nocardioides* and *norank_c__Acidobacteria* recorded the greatest changes due to the addition of soft rock in the treatments. The addition of different proportions of soft rock considerably reduced the abundance of *Bacillus* in all treatments. Under treatment C1, *norank_c__Acidobacteria* recorded a noticeable increase in abundance; *Nocardioides* abundance significantly increased in treatment C2; and the abundance of *norank_f__Anaerolineaceae* and *norank_c__Acidobacteria* considerably increased in C3. The results of double clustering showed that the community structure and species composition of C1 and C2 treatments were similar, while CK and C3 treatments were clustered separately. *Bacillus*, *Pseudomonas*, *Lysinimonas*, *Microvirga*, and *Enterococcus* were classified into one group, indicating that their structures are similar; the addition of soft rock reduced their species abundance. *Pseudarthrobacter* was divided into a separate group, as were *norank_o__JG30-KF-CM45* and *norank_c__Acidobacteria*. As the addition of soft rock also resulted in an increase in abundance of n*orank_f__Anaerolineaceae*, *Nocardioides*, *Massilia*, *norank_p__Saccharibacteria* and *Sphingomonas*, these species were also divided into a separate group. Finally, the remaining seven species were grouped together.

### Alpha diversity analysis

Bacteria are the most abundant group of soil microorganisms, generally accounting for 70–90% of soil microorganisms^13^. Bacteria have the most abundant genetic diversity, they can effectively promote the decomposition of organic matter and the release of nutrients, participate in the carbon and nitrogen cycles, and maintain the energy flow and material cycle of the ecosystem^29^. Compared to the CK treatment, the addition of soft rock in treatments C1, C2 and C3 promoted bacteria diversity, richness and evenness, resulting in noticeable increases in the Shannon index (7.36–9.36%), the Ace index (9.79–16.48%), the Chao index (10.29–17.64%) and the Shannoneven index (3.95–6.58%) (Table 3). Among these results, the richness index decreased with an increase of the proportion of soft rock, and the diversity index and evenness index were higher with treatment C2. Results for soil carbon content (TOC, POC, DOC, ROC and SMBC) increased with different degrees after the addition of soft rock (Table 1), suggesting an abundance of energy substances for bacteria growth and reproduction. Particle composition analysis of a soft rock:sand compound soil undertaken by Guo et al.^23^ concluded that the addition of soft rock improved the texture of the sandy soil, increased aeration and agglomeration, and provided a favorable external environment for bacterial metabolism. Compared with degraded sandy land, the investigation by Zhang et al.^30^ highlighted that vegetation restoration can promote an increase in soil carbon and nitrogen nutrients in a sandy land, as well as improving the genetic diversity of soil bacterial communities, findings that are in accordance with our results. Coverage indicates the degree of coverage detected by the gene library, and a larger value indicates a more reasonable sequencing result. Coverage results in our study indicate that the coverage of each treatment was over 98%, including most bacterial communities, and the sequencing results were reasonable. Multiple regression analysis of soil organic carbon component content and diversity index showed that POC was significantly positively correlated with Shannon index (P < 0.01). At the same time, Ace and Chao indexes were significantly positively correlated with POC and ROC content (Table 4). This finding suggests that an increase in POC and ROC content could effectively promote bacteria diversity and richness. ROC/TOC in each treatment recorded a significant difference compared with ROC. ROC/TOC in C2 was the largest (Table 2), indicating that ROC changes were sensitive and closely related to the diversity parameters of bacteria.

**Table 3:** Soil bacterial diversity parameters under different compound ratios of soft rock:sand.

Treatments	Community diversity		Community richness		Community evenness		Coverage (%)
	Shannon	Simpson	Ace	Chao	Shannoneven	Simpsoneven	
CK	5.71 ± 0.20 b	0.0127 ± 0.0025 a	2360 ± 146 b	2352 ± 166 b	0.76 ± 0.02 b	0.04 ± 0.01 a	98.63 ± 0.13 a
C1	6.13 ± 0.13 a	0.0103 ± 0.0025 a	2749 ± 27 a	2767 ± 51 a	0.79 ± 0.01 a	0.04 ± 0.01 a	98.50 ± 0.31 a
C2	6.25 ± 0.20 a	0.0081 ± 0.0042 a	2669 ± 56 a	2702 ± 38 a	0.81 ± 0.03 a	0.07 ± 0.02 a	98.42 ± 0.35 a
C3	6.18 ± 0.10 a	0.0071 ± 0.0016 a	2591 ± 69 a	2594 ± 67 a	0.81 ± 0.01 a	0.07 ± 0.01 a	98.59 ± 0.09 a

CK, the volume ratio of soft rock to sand is 0:1; C1, the volume ratio of soft rock to sand is 1:5; C2, the volume ratio of soft rock to sand is 1:2; C3, the volume ratio of soft rock to sand is 1:1.

**Table 4:** Correlation coefficient test after multiple regression analysis of organic carbon components and bacterial diversity parameters.

Index	TOC	POC	DOC	ROC	SMBC
Shannon	0.5363	0.7466**	0.3446	0.6579*	0.5837*
Simpson	− 0.1900	− 0.5041	− 0.3369	− 0.2603	− 0.4104
Ace	0.6058*	0.7748**	0.4610	0.7316**	0.6856*
Chao	0.5752	0.7466**	0.4279	0.7300**	0.6536*
Shannoneven	0.3171	0.1320	− 0.1871	0.1418	0.4024
Simpsoneven	0.0450	0.3691	− 0.0462	0.2878	0.2760

*TOC* soil total organic carbon, *POC* particulate organic carbon, *DOC* dissolved organic carbon, *ROC* oxidized organic carbon, *SMBC* soil microbial biomass carbon.

* and ** indicate significant correlations of 5% and 1%, respectively.

### Effects of organic carbon components on bacterial community structure

**Figure 3:**
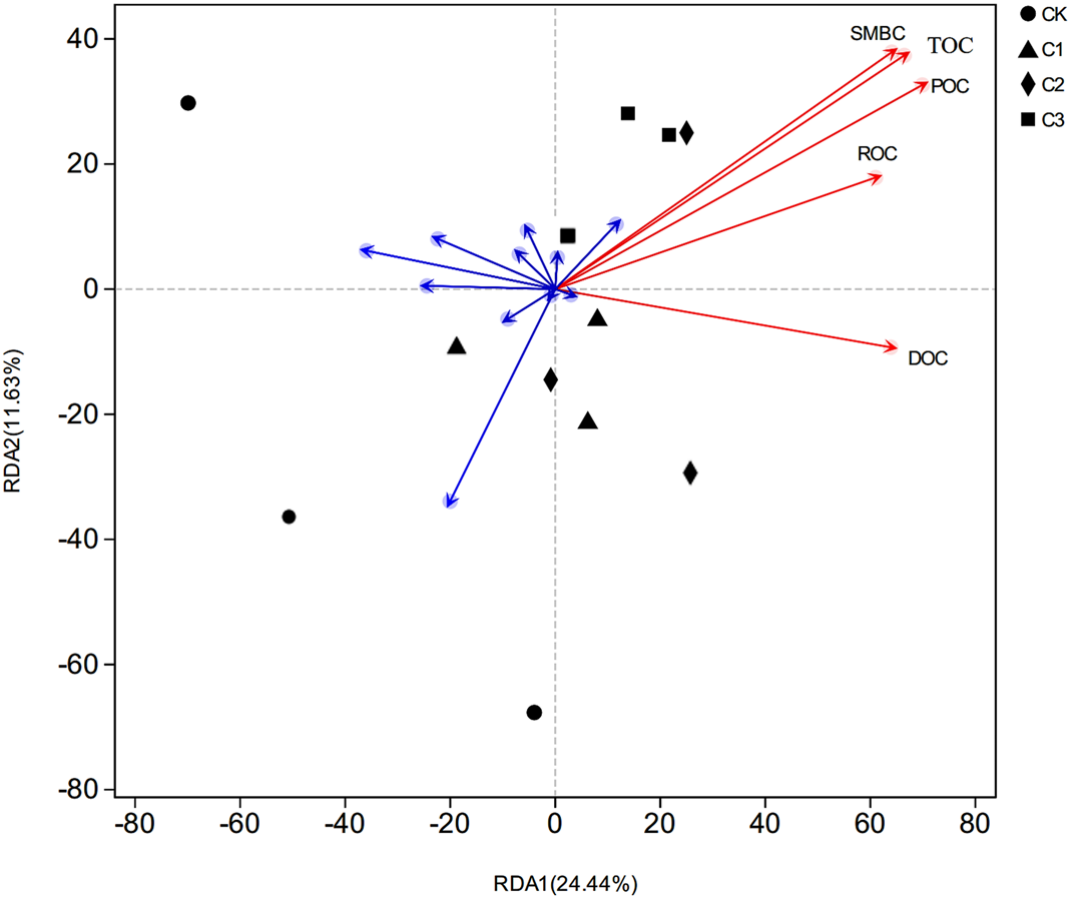
Redundant analysis of organic carbon components and bacterial community structure in soils with different mixing ratios of soft rock:sand. CK, the volume ratio of soft rock to sand is 0:1; C1, the volume ratio of soft rock to sand is 1:5; C2, the volume ratio of soft rock to sand is 1:2; C3, the volume ratio of soft rock to sand is 1:1. *TOC* soil total organic carbon; *POC* particulate organic carbon; *DOC* dissolved organic carbon; *ROC* oxidized organic carbon; *SMBC* soil microbial biomass carbon.

Changes in the soil bacterial community structure mainly depend on the physical and chemical properties of the soil, such as pH, porosity, colloids, texture and organic matter content^31^. In our study, soils with different ratios of soft rock:sand were used to perform redundant analysis between the organic carbon component and the bacterial community structure. Our results showed that POC had the most significant effect on bacterial community structure (r = 0.8432, P < 0.05), explaining 24.44% of the community change (Fig. 3). Because POC accounts for the fraction of organic carbon that was combined with the sand component of the soil^32^, the addition of soft rock promoted the composition of sandy soil aggregates which had a greater impact on soil structure^19^, thereby greatly changing the bacterial community structure. Our results, similar to those of Liu et al.^33^, also indicated that TOC had a significant effect on bacterial community structure (r = 0.8294, P < 0.05), explaining 11.63% of the community change. Soil microorganisms, as an indispensable active part in the biosphere, participate in processes such as degradation of animal and plant residue, nutrient cycling and balance, and their microbial biomass carbon is an essential indicator of soil nutrient conversion cycles and energy flow^34^. Our results showed that SMBC also had a significant impact on the bacterial community structure (r = 0.8086, P < 0.05), explaining 9.56% of the community changes. All components of organic carbon explained 55.74% of community changes, having an order of influence of POC > TOC > SMBC > DOC > ROC. As the bacterial diversity index and the richness index significantly increased (Table 3), the carbon content of microbial residue in the soil increased. Soft rock improved the sandy soil environment, promoting the development of water, fertilizer, gas and heat coordination ability^18^. Along the direction of the second axis in redundant analysis (Fig. 3), bacterial community treated by CK was separated from other treatments, indicating that the addition of soft rock had a significant influence on bacterial community structure. However, community staggered distributions of C1 and C2 indicated that the community composition of the two treatments had a high level of similarity.

### Relationship between crop yield and bacterial community structure

**Figure 4:**
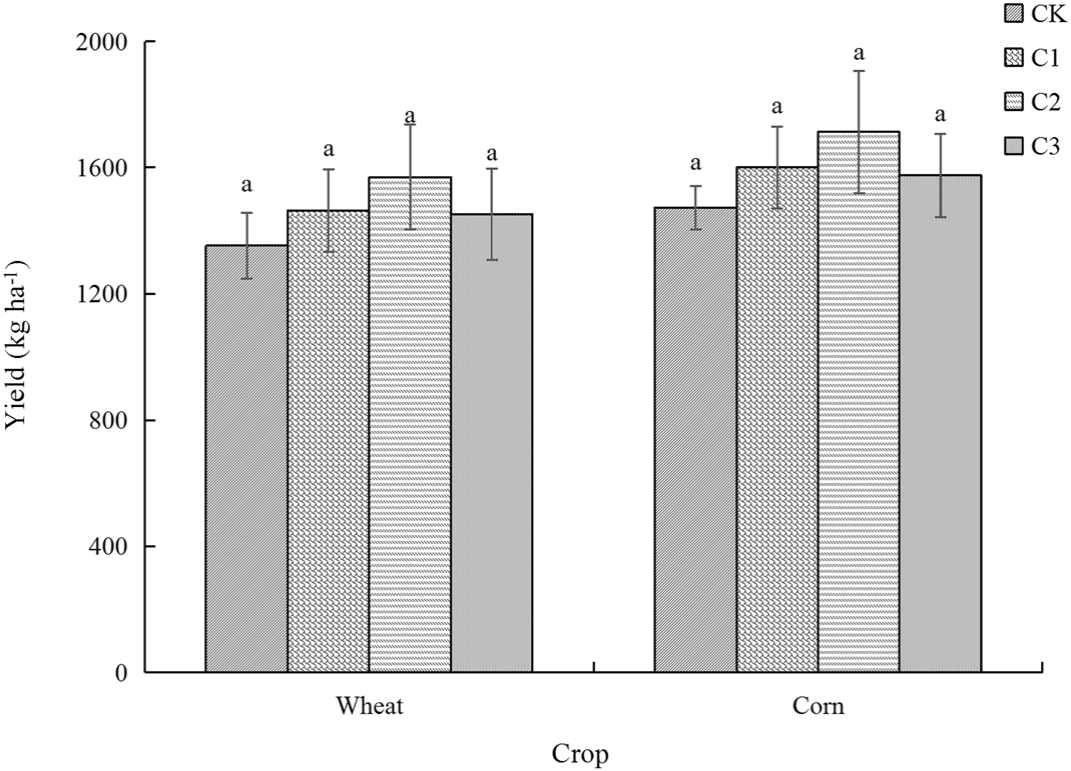
Crop yield in the soft rock:sand compound soil. CK, the volume ratio of soft rock to sand is 0:1; C1, the volume ratio of soft rock to sand is 1:5; C2, the volume ratio of soft rock to sand is 1:2; C3, the volume ratio of soft rock to sand is 1:1. *TOC* soil total organic carbon; *POC* particulate organic carbon; *DOC* dissolved organic carbon; *ROC* oxidized organic carbon; *SMBC* soil microbial biomass carbon. The lowercase letters in the figure indicate significant differences between the different treatments at the 5% level.

**Figure 5:**
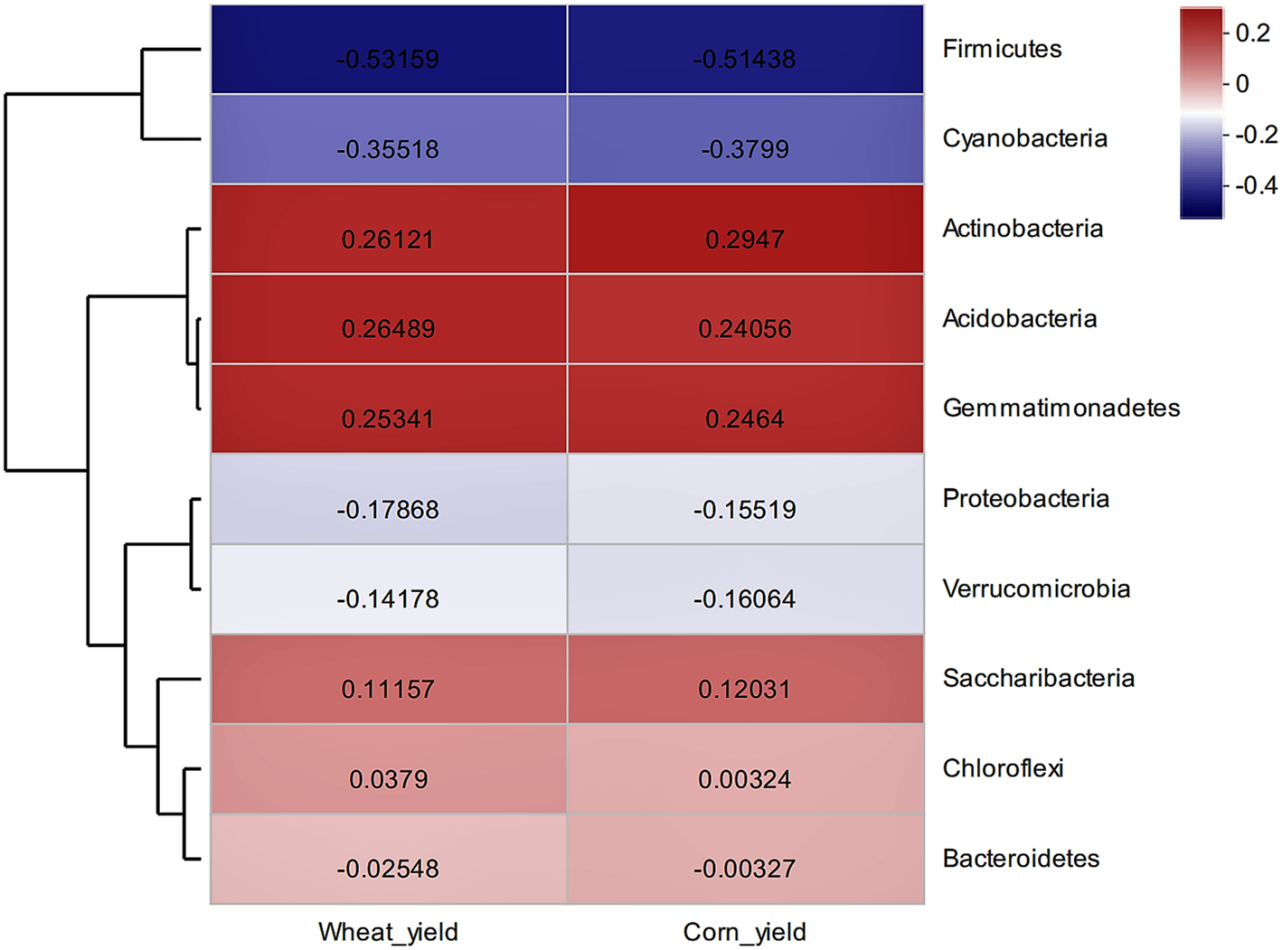
The relationship between crop yield and bacterial community structure in the soft rock:sand compound soil. The image was created with R (pheatmap package).

Two crops (wheat and corn) were mainly grown on the soft rock: sand compound soil. Crop yield in our study indicated that wheat and corn recorded the same change trend under the different treatments. Compared with the CK treatment, wheat and corn yields all recorded an increase under the different treatments: 8.28% and 8.70% in C1; 16.05% and 16.30% in C2; and 7.40% and 7.00% in C3, respectively, but there was no significant difference (P > 0.05) (Fig. 4). Because the saturated hydraulic conductivity of the composite soil rapidly decreased as the proportion of soft rock increased^35^. When the mixing ratio of soft rock:sand was in the range of 1:5–1:2, the tendency of the saturated hydraulic conductivity to decrease rapidly declined^35^. In addition, with an increase in the proportion of soft rock, capillary porosity continuously increased, resulting in an increase in water retention and water holding capacity of the soil, providing favorable water conditions and texture structure for crop growth^23,36^. Although correlation analysis between crop yield and bacterial community abundance showed that there was no significant correlation between bacterial abundance and crop yield, bacterial community structure had a certain influence (Fig. 5). Clustering analysis divided bacteria into four categories, with *Firmicutes* and *Cyanobacteria* in one category; *Actinobacteria*, *Acidobacteria* and *Gemmatimonadetes* in one category; *Proteobacteria* and *Verrucomicrobia* in one category; and *Saccharibacteria*, *Chloroflexi* and *Bacteroidetes* in one category (Fig. 5). As the three dominant bacteria (*Proteobacteria*, *Actinobacteria* and *Chloroflexi*) were distributed in different categories (Figs. 1; 5), this indicates that decreasing the abundance of *Proteobacteria* and increasing the abundance of *Actinobacteria* and *Chloroflexi* can promote an increase in crop yield.

## Conclusions

The addition of soft rock plays a certain role in promoting the organic carbon pool and bacterial diversity in a sandy soil. Our results indicated that TOC, POC and ROC contents in C1, C2 and C3 treatments were higher than those in the CK treatment. DOC and SMBC content in C2 did not noticeably differ from those in CK, and only treatment C3 recorded a significant increase in SMBC content. No significant differences were recorded between ROC treated by C1, C2 and C3, however ROC/TOC noticeably differed between treatments, among which ROC/TOC treated with C2 was the largest. Compared with the CK treatment, bacterial richness index, diversity index and evenness index in C1, C2 and C3 treatments noticeably increased, promoting the growth and reproduction of bacteria. Among these, the bacterial community structure of C1 and C2 were relatively similar; the CK treatment and C3 were clustered separately. Although the three dominant bacteria were *Proteobacteria*, *Actinobacteria*, and *Chloroflexi*, the abundance of *Actinobacteria* declined in C3 and increased in C1 and C2. Our results indicated that a composition ratio between 1:5 and 1:2 (soft rock:sand) was most advantageous; changes of organic carbon components were relatively sensitive and the bacterial diversity parameters were large. Our results also indicated that POC, TOC and SMBC were the main factors affecting bacterial community structure changes. With a soft rock:sand ratio between 1:5 and 1:2, crop yield was recorded to be higher, related to the community structure distribution of the dominant bacteria. Our results provide new information which can be used when implementing future management decisions for improving sandy land utilizing soft rock, as well as providing guidance for the management of degraded and unused land in these areas.

## Materials and methods

### Overview of the test site

A long-term study of compound soil was undertaken in Fuping county, Shaanxi province (109° 11′ E, 34° 42′ N), in the transition zone between the Guanzhong plain and the northern Shaanxi plateau. Elevation in the study area is 380.8–1421.5 m, and this area belongs to the warm temperate zone, whereshows continental monsoon climate. Annual total radiation is 5187.4 MJ m^−2^, annual average sunshine hours are about 2389.6 h, annual average temperature is 13.1 °C, and the annual average precipitation is 527.2 mm.

### Experiment design

Soft rock in the study area is characterized by quartz, montmorillonite, feldspar, calcite, illite, kaolinite and dolomite minerals. The main chemical constituents of the soft rock are SiO_2_ (65% by mass), Al_2_O_3_ (14% by mass), Fe_2_O_3_ and CaO (21% by mass). Sand is mainly composed of quartz (SiO_2_; 82% by mass), with feldspar (10% by mass), kaolinite (4% by mass), calcite (2% by mass) and amphibole (2% by mass). By using a long-term location study, soil conditions in a mixed layer in the Mu Us sandy were simulated. In the experimental plot, a layer composing soft rock and sand was established (30 cm) on top of a layer (30–70 cm) of aeolian sandy soil. The experiment was established in 2009, and four treatments with a soft rock:sand ratio of 0:1 (CK), 1:5 (C1), 1:2 (C2) and 1:1 (C3) were selected. Each treatment was repeated three times in a total of 12 trial plots. The experimental field was corn (Jincheng 508)-wheat (Xiaoyan 22), which was made by two crops a year, all of which were artificially sown^23^. Fertilizers and topsoil were properly mixed through deep tillage for experimental plots. Fertilizers used in the experiment were urea (including N 46.4%), diammonium phosphate (including N 16%, containing P_2_O_5_ 44%) and potassium sulfate (including K_2_O 52%). Fertilizers were added with the rates of 255 kg hm^−2^ (N), 180 kg hm^−2^ (P_2_O_5_) and 90 kg hm^−2^ (K_2_O). The experimental field was adequately irrigated, and the irrigation volume was 4000 m^3^ ha^−1^ annually during the study years.

### Soil sample collection

After wheat was harvested in May 2019, five soil samples in the upper soil layer (of 0–30 cm) were collected from each plot and combined to form a composite sample. Animal and plant residues were removed from the soil samples before being were passed through a 1 mm sieve and divided into two sub-samples. One sub-sample was stored at − 80 °C freezer prior to high-throughput sequencing, and the other sub-sample was naturally air-dried and ground before being passed through a 0.25 mm sieve prior to carbon index determination.

### Determination methods

SOC and POC were both analyzed using the potassium dichromate-concentrated sulfuric acid external heating method^37^. DOC was measured using the deionized water extraction method; the water-soil ratio was 10:1 and the extract was tested using a TOC analyzer (Multi N/C 3100, Analytik Jena AG, Germany)^38^. ROC was analyzed using the potassium permanganate oxidation method, and colorimetry was performed on an ultraviolet spectrophotometer (UV-1900iUV-1900, Shimadzu, Japan)^39^. SMBC was determined using the chloroform fumigation-extraction method^40^.

DNA was extracted using the following method: bacterial DNA was extracted from 0.5 g soil samples stored at − 80 °C, as per the soil DNA kit instructions (Omega bio-tek, Norcross, GA, USA). DNA purity and concentration were determined using a NanoDrop 2000 spectrophotometer (Thermo Scientific, Wilmington, USA), and DNA integrity was detected using 1% agarose gel electrophoresis.

PCR amplification and high-throughput sequencing was undertaken by selecting 338 F (5′-ACTCCTACGGGAGGCAGCAG-3′) and 806R (5′-GGACTACHVGGGTWTCTAAT-3′) general primers in the V3-V4 region of 16S rRNA gene^41^. The formal PCR test used TransGen AP221-02: TransStart Fastpfu DNA Polymerase, 20 µL reaction system: 5 × FastPfu Buffer 4 µL, 2.5 mM dNTPs 2 µL, Forward Primer (5 µM) 0.8 µL, Reverse Primer (5 µM) 0.8 µL, FastPfu Polymerase 0.4 µL, BSA 0.2 µL, Template DNA 10 ng, Supplement ddH_2_O to 20 µL. The PCR reaction procedure was as follows: 95 °C for 3 min, 30 cycles, 95 °C for 30 s, 55 °C for 30 s, 72 °C for 45 s, 72 °C for 10 min. The amplification product was detected using 2% agarose gel electrophoresis. After measuring the concentration of the purified product, the equimolar number was mixed. Sequencing was performed using a Illumina MiSeq platform (Illumina, San Diego, USA) according to the standard protocol of Majorbio Bio-Pharm Technology Co. Ltd. (Shanghai, China).

### Data analysis

Each organic carbon component was analyzed using Duncan multiple comparison and analysis of variance in Microsoft Excel 2010 and SPSS 20.0 (IBM, Stanford, USA).

Paired-end (PE) reads obtained by Miseq sequencing were initially spliced according to their overlap relationship, and quality control and filtration were conducted for sequence quality. Effective sequences were distinguished according to the barcode and primer sequences at both ends of the sequence, and the sequence direction was corrected. The RDP classifier Bayesian algorithm was used to classify the 97% similar level operational taxon units (OTU) of the representative sequence, and the community composition of each sample was counted at the Phylum and Genus taxonomic level. Species Alpha diversity analysis was performed by Mothur software (Version V.1.30.1) based on OTU. Multiple regression analysis of organic carbon composition and diversity index was carried out through SPSS 20.0 software. Redundancy analysis (RDA) employed rda in the vegan package of R language for mapping. Sequencing data were analyzed using Majorbio I-Sanger (https://www.i-sanger.com), a free online cloud platform.
